# Risks of large-scale use of systemic insecticides to ecosystem functioning and services

**DOI:** 10.1007/s11356-014-3277-x

**Published:** 2014-07-19

**Authors:** Madeleine Chagnon, David Kreutzweiser, Edward A.D. Mitchell, Christy A. Morrissey, Dominique A. Noome, Jeroen P. Van der Sluijs

**Affiliations:** 1 Département des sciences biologiques, Université du Québec à Montréal, Case Postale 8888, Succursale Centre-Ville, Montréal, Québec H3C 3P8 Canada; 2 Canadian Forest Service, Natural Resources Canada, 1219 Queen St. East, Sault Ste. Marie, Ontario P6A 2E5 Canada; 3 Laboratory of Soil Biology, University of Neuchâtel, Rue Emile Argand 11, 2000 Neuchâtel, Switzerland; 4 Jardin Botanique de Neuchâtel, Chemin du Perthuis-du-Sault 58, 2000 Neuchâtel, Switzerland; 5 Department of Biology and School of Environment and Sustainability, University of Saskatchewan, 112 Science Place, Saskatoon, Saskatchewan S7N 5E2 Canada; 6 Task Force on Systemic Pesticides, 46, Pertuis-du-Sault, 2000 Neuchâtel, Switzerland; 7 Kasungu National Park, c/o Lifupa Conservation Lodge, Private Bag 151, Lilongwe, Malawi; 8 Environmental Sciences, Utrecht University, Heidelberglaan 2, 3584 CS Utrecht, The Netherlands; 9 Centre for the Study of the Sciences and the Humanities, University of Bergen, Postboks 7805, N-5020 Bergen, Norway

**Keywords:** Ecosystem services, Soil ecosystem, Neonicotinoids, Pollinators, Freshwater, Rice paddies

## Abstract

Large-scale use of the persistent and potent neonicotinoid and fipronil insecticides has raised concerns about risks to ecosystem functions provided by a wide range of species and environments affected by these insecticides. The concept of ecosystem services is widely used in decision making in the context of valuing the service potentials, benefits, and use values that well-functioning ecosystems provide to humans and the biosphere and, as an endpoint (value to be protected), in ecological risk assessment of chemicals. Neonicotinoid insecticides are frequently detected in soil and water and are also found in air, as dust particles during sowing of crops and aerosols during spraying. These environmental media provide essential resources to support biodiversity, but are known to be threatened by long-term or repeated contamination by neonicotinoids and fipronil. We review the state of knowledge regarding the potential impacts of these insecticides on ecosystem functioning and services provided by terrestrial and aquatic ecosystems including soil and freshwater functions, fisheries, biological pest control, and pollination services. Empirical studies examining the specific impacts of neonicotinoids and fipronil to ecosystem services have focused largely on the negative impacts to beneficial insect species (honeybees) and the impact on pollination service of food crops. However, here we document broader evidence of the effects on ecosystem functions regulating soil and water quality, pest control, pollination, ecosystem resilience, and community diversity. In particular, microbes, invertebrates, and fish play critical roles as decomposers, pollinators, consumers, and predators, which collectively maintain healthy communities and ecosystem integrity. Several examples in this review demonstrate evidence of the negative impacts of systemic insecticides on decomposition, nutrient cycling, soil respiration, and invertebrate populations valued by humans. Invertebrates, particularly earthworms that are important for soil processes, wild and domestic insect pollinators which are important for plant and crop production, and several freshwater taxa which are involved in aquatic nutrient cycling, were all found to be highly susceptible to lethal and sublethal effects of neonicotinoids and/or fipronil at environmentally relevant concentrations. By contrast, most microbes and fish do not appear to be as sensitive under normal exposure scenarios, though the effects on fish may be important in certain realms such as combined fish-rice farming systems and through food chain effects. We highlight the economic and cultural concerns around agriculture and aquaculture production and the role these insecticides may have in threatening food security. Overall, we recommend improved sustainable agricultural practices that restrict systemic insecticide use to maintain and support several ecosystem services that humans fundamentally depend on.

## Introduction

Other papers in this special issue have shown that neonicotinoid insecticides and fipronil are presently used on a very large scale (e.g., Simon-Delso et  al. 2014, this issue) and are highly persistent, and repeated application can lead to buildup of environmental concentrations in soils. They have high runoff and leaching potential to surface and groundwaters and have been detected frequently in the global environment (Bonmatin et  al. 2014, this issue). Evidence is mounting that they have direct and indirect impacts at field realistic environmental concentrations on a wide range of nontarget species, mainly invertebrates (Pisa et  al. 2014, this issue) but also on vertebrates (Gibbons et  al. 2014, this issue). Although studies directly assessing impacts to ecosystem functions and services are limited, here we review the present state of knowledge on the potential risks posed by neonicotinoids and fipronil.

The concept of ecosystem services is widely used in decision making in the context of valuing the service potentials, benefits, and use values that well-functioning ecosystems provide to humans and the biosphere (Spangenberg et  al. 2014a, b). Ecosystem services were initially defined as “benefits people obtain from ecosystems” as popularized by the United Nations Environment Program (UNEP 2003) and the Millennium Ecosystem Assessment (MEA 2003, 2005). They are seen as critical to the functioning of the Earth’s life support system, which consists of habitats, ecological systems, and processes that provide services that contribute to human welfare (Costanza et  al. 1997). Under the MEA framework (among others), ecosystem services have been categorized into provisioning services (e.g., food, wood, fiber, clean water), regulating services (e.g., climate control, detoxification, water purification, pollination, seed dispersal, pest and disease regulation, herbivory, and weed control), supporting services (e.g., soil formation, nutrient cycling, pollination, soil quality, food web support, waste treatment, and remediation), and cultural services (e.g., recreation, esthetic, or spiritual value).

The wide application of neonicotinoid systemic pesticides, their persistence in soil and water, and potential for uptake by crops and wild plants expose a wide range of species, which are important in providing valuable ecosystem services. This paper addresses the risks to ecosystem functioning and services from the growing use of systemic neonicotinoid and fipronil insecticides used in agricultural and urban settings. Here, we focus on ecosystem services provided by terrestrial soil ecosystem functions, freshwater ecosystem functions, fisheries, biological pest control, and pollination, in addition to reviewing the overall threats of these systemic insecticides to food security.

## Terrestrial soil ecosystem functions

### Soil ecosystem services and biodiversity

Terrestrial ecosystems are known to provide a complex range of essential ecosystem services involving both physical and biological processes regulated by soils. Soils support physical processes related to water quality and availability such as soil structure and composition (e.g., porosity) to facilitate movement of water to plants, to groundwater aquifers, and to surface water supplies. Water quality is improved by filtration through clean soils that can remove contaminants and fine sediments. As water flows through soils, it interacts with various soil matrices absorbing and transporting dissolved and particulate materials including nutrients and other life-supporting elements to plants and microorganisms. Soils further provide stream flow regulation and flood control by absorbing and releasing excess water.

Many of the soil ecosystem services are biologically mediated, including regulation and cycling of water and nutrients, the facilitation of nutrient transfer and translocation, the renewal of nutrients through organic and waste matter breakdown, elemental transformations, soil formation processes, and the retention and delivery of nutrients to plants (Swift et  al. 2004; Dominati et  al. 2010; Robinson et  al. 2013). Plants, in turn, provide food, wood, and fiber to support human infrastructure and natural habitats, while improving soil retention and erosion control. Over the long term, they also provide raw materials for consumption such as peat for fuel and horticultural substrates and ornamental plants and flowers for decoration. Further services include the biological control of pests and diseases through provision of soil conditions and habitats for beneficial species and natural enemies of pests, the sequestration and storage of carbon through plant growth and biomass retention, and the detoxification of contaminants through sorption, immobilization, and degradation processes.

Many of the biologically mediated soil ecosystem services listed above require the inputs and activities of interacting diverse and functional biological communities (Swift et  al. 2004; Lavelle et  al. 2006; Barrios 2007). Biodiversity conservation itself can be considered as an important ecosystem service (Dale and Polasky 2007; Eigenbrod et  al. 2010), following on the earlier concept that biodiversity serves as a form of insurance against the loss of certain species and their ecological function through species redundancy (Naeem and Li 1997; Yachi and Loreau 1999). Biodiversity has been shown to be positively related to ecological functions that support ecological services (Benayas et  al. 2009). The stability of soil ecosystems has been linked to biodiversity and especially the relative abundances of keystone species or functional groups that underpin the soil food web structure or that facilitate specialized soil processes (de Ruiter et  al. 1995; Brussaard et  al. 2007; Nielsen et  al. 2011).

Natural soils are a reservoir of diverse and complex biological communities. Organisms range from body sizes in millimeters (macrofauna, macroflora) to cell or body sizes in micrometers (mesofauna, microfauna, microflora). Key taxa include macroarthropods (e.g., ground beetles, ants, termites), earthworms, mites, collembolans, protozoans, nematodes, bacteria, and fungi. The activity of these biota and interactions among them condition ecosystem processes on which many ecosystem services depend (Barrios 2007). For example, earthworms have a large impact on organic matter dynamics, nutrient cycling, and soil properties. Earthworms break down plant litter into nutrient-rich organic matter for other consumers and contribute to the mixing of organic matter in soils. They produce casts, mucilages, and other nutrient-rich excretions that contribute to soil fertility and biogeochemical cycling (Beare et  al. 1995). Their burrowing activity increases soil porosity and aeration, facilitates water and nutrient transfer, and reduces soil compaction (Edwards and Bohlen 1996). While earthworms play a key role in soil organic matter dynamics, the decomposition and mineralization of organic matter is a complex process that is facilitated by the activities and interactions among diverse biotic communities including other invertebrates, protists, bacteria, and fungi (Swift et  al. 2004). These biota-mediated soil processes occur at a scale of centimeters to decimeters by individuals and populations, and the accumulation of these processes over space and time creates a continuous process from which soil properties and services arise to local and regional landscape scales (Lavelle et  al. 2006).

A further example of ecosystem services is the biologically mediated nitrogen cycling in soils. Nitrogen (N) is essential for plant growth, and plants convey many of the services derived from soils. Macro- and meso-invertebrates initiate decomposition of soil organic matter by fragmentation, ingestion, and excretion to release organic N which is subsequently mineralized by highly specialized microbial groups to plant-available forms of inorganic N. Available N pools in soils are also greatly enhanced by nitrogen-fixing microorganisms that convert atmosphere N to plant-available N through root nodule symbioses in plants, especially legumes. Inorganic N can also be taken up by soil microbes, assimilated into biomass, and incorporated into the soil organic N pool (immobilization), which is available for further cycling (Brady and Weil 1996; Brussaard et  al. 1997; Barrios 2007). The excess of N is a major cause of soil and water eutrophication with consequences on biodiversity (Vitousek et  al. 1997), and therefore, loss of N through denitrification is a another valuable ecosystem service provided by wetlands and floodplain forest soils (Shrestha et  al. 2012).

### Impacts of neonicotinoid insecticides on soil ecosystem services

Given that many of the ecosystem services of soils are biologically mediated, and pesticides can cause depletion or disruption of nontarget biotic communities in soils, it follows that pesticides can pose risks to soil ecosystem processes and services. Effects of pesticides in soils can range from direct acute and chronic toxicity in organisms to many sublethal or indirect effects on behavior, functional roles, predator-prey relationships, and food web dynamics. Any or all of these can occur at the organism, population, or community levels and, therefore, may impact soil biodiversity or ecosystem stability (Edwards 2002). Since soil biodiversity is related to ecological functions that support ecological services (Benayas et  al. 2009), pesticide-induced disruptions to biodiversity and ecological function could impair ecosystem services derived from soils (Goulson 2013). Impacts on soil biodiversity and their implications for ecosystem function have been demonstrated for other pesticides affecting microbial (Johnsen et  al. 2001) and invertebrate (Jansch et  al. 2006) communities, and the same risks are likely to arise from neonicotinoid insecticides in soils. Neonicotinoids can persist in soils for several years (Goulson 2013; Bonmatin et  al. 2014, this issue) and can cause significant adverse effects on key soil organisms at environmentally realistic concentrations (Pisa et  al. 2014, this issue) and, therefore, have the potential to pose a risk to soil ecosystem services.

While the link between adverse effects on organisms and ecological function or services in soils is theoretically sound, empirical evidence of effects on soil ecosystem services from neonicotinoid insecticides is sparse, partly because its large-scale use started only a decade ago. In our review of the literature, we found only a few studies that reported the effects of neonicotinoids on soil organism function with implications for ecosystem services. Peck (2009a, b) assessed the impacts of the neonicotinoid, imidacloprid, applied to turfgrass for scarab beetle control and found direct and indirect long-term effects on some arthropods and suggested negative implications (although not empirically tested) for soil nutrient cycling and natural regulation of pests. In laboratory microcosms, Kreutzweiser et  al. (2008a, 2009) tested the effects of imidacloprid in the leaves from systemically treated trees on the breakdown of autumn-shed leaves by litter dwelling earthworms over a 35-day exposure period. At realistic field concentrations, the leaf-borne residues of imidacloprid were not directly toxic to earthworms, but did cause feeding inhibition that resulted in a significant reduction in leaf litter breakdown. They further demonstrated that this effect was due to sublethal toxic effects, not avoidance behavior (Kreutzweiser et  al. 2009). When imidacloprid was added directly to terrestrial microcosms to simulate a soil injection method for treating trees, a similar effect was detected with significantly reduced breakdown of leaf litter by earthworms at ambient litter concentrations of 7 mg/kg and higher (Kreutzweiser et  al. 2008b). Taken together, these studies demonstrated that when imidacloprid is applied as a systemic insecticide for the control of wood-boring insects in trees, residual imidacloprid in autumn-shed leaves poses risk of reduced leaf litter breakdown through a feeding inhibition effect on earthworms, and this has negative implications for organic matter dynamics in soils. A similar effect would presumably occur in the breakdown of other imidacloprid-bearing plant litter in other soils, including agricultural but, to our knowledge, this has not been tested directly. Other effects of neonicotinoids on earthworm behavior that may further influence ecological processes in soils (e.g., burrowing behavior) are reviewed in Pisa et  al. (2014, this issue).

Soil microbial communities have also been affected by imidacloprid, which can affect leaf litter decomposition. Although imidacloprid did not inhibit microbial decomposition of autumn-shed leaves of ash trees (*Fraxinus* spp.) (Kreutzweiser et  al. 2008b), microbial decomposition of leaves from maple (*Acer saccharum*) trees was significantly inhibited at concentrations expected from systemic treatments to control wood-boring insects (Kreutzweiser et  al. 2008a). The authors offer suggestions for observed differences in effects among tree species. Regardless of differences between studies, the data indicate that imidacloprid residues in leaf material have the potential to interfere with microbial decomposition of leaf litter, with implications for organic matter breakdown and nutrient cycling.

Others have assessed the effects of imidacloprid on microbial activity in agricultural soils after treated seed applications. Singh and Singh (2005a) measured microbial enzyme activity as an indicator of population level effects and found that imidacloprid in soils after seed treatment had stimulatory effects on microbial enzyme activity for up to 60 days. In the same set of experiments, they also measured available N in soils and reported increased available N (Singh and Singh 2005b). In a further study at the same site, Singh and Singh (2006) found increased nitrate-N but decreased ammonium, nitrite-N, and nitrate reductase enzyme activity in soils in which imidacloprid-coated seeds had been planted. Tu (1995) added imidacloprid to sandy soils and reported decreased fungal abundance and short-term decreases in phosphatase activity but no measurable effects on nitrification or denitrification rates. Ingram et  al. (2005) reported no inhibition of microbial urease activity by imidacloprid in turfgrass soil or sod. Similarly, Jaffer-Mohiddin et  al. (2010) found no inhibition, and some stimulation, of amylase and cellulase activity in soils under laboratory conditions. Ahemad and Khan (2012) measured decreased activity and plant growth promoting traits of a N-fixing bacterium, *Rhizobium* sp., isolated from pea nodules of plants exposed to imidacloprid in soils, but only at three times the recommended application rate (no significant effects at the recommended rate). Overall, these studies demonstrate that neonicotinoids can induce measurable changes in soil microbial activity but the effects are often stimulatory, short-term, and of little or no measurable consequence to soil nutrient cycling. The reported microbial responses have been attributed to inductive adaptation as microbes assimilate or mineralize components of the imidacloprid molecule (Singh and Singh 2005a), essentially a biodegradation process (Anhalt et  al. 2007; Liu et  al. 2011; Zhou et  al. 2013; Wang et  al. 2013).

By contrast, at least two other studies have reported adverse or negative effects of neonicotinoids on soil microbial communities and their function. Yao et  al. (2006) reported significantly inhibited soil respiration at field realistic concentrations of acetamiprid. Cycon et  al. (2013) found measurable changes in soil community structure and diversity, and that these were generally found in conjunction with reduced soil metabolic activity at or near realistic field rates of imidacloprid. It is possible that community level changes associated with the neonicotinoid exposure may facilitate the adaptive responses in functional parameters listed above.

### Conclusions on soils as ecosystem services

Given that many soil ecosystem services are dependent on soil organisms, that neonicotinoid insecticides often occur and can persist in soils, and that their residues pose a risk of harm to several key soil invertebrates, neonicotinoids have the potential to cause adverse effects on ecosystem services of soils. From a theoretical perspective and based on findings from studies of better-studied pesticides, the potential for neonicotinoid impacts on soil ecosystem services appears to be high but there are few empirical studies that have tested these effects. From the few studies available, it appears that invertebrate-mediated soil processes are at greater risk of adverse effects from neonicotinoid residues than are microbial-mediated processes.

One issue that remains elusive is the degree to which soil biological communities can absorb pesticide impacts before ecosystem function, and ultimately, the delivery of services is measurably impaired at a local or regional scale. Studies are conflicting with regard to the degree of functional redundancy and resilience inherent in soil and other biological communities that are rich in diversity. Swift et  al. (2004) review the impacts of agricultural practices, including the use of pesticides, on the relationship between biodiversity and ecosystem function and show that some changes in biological communities can be harmful to ecosystem function while others are functionally neutral. They suggest that microbial communities have a high degree of functional redundancy and resilience to impacts on their functional role in soil organic matter processing. On the other hand, reductions in highly specialized taxa with unique or critical roles in an important ecosystem function such as decomposition and nutrient cycling can measurably impact the delivery of ecosystem services (Barrios 2007). Earthworms could be categorized as such, and since adverse effects on earthworms have been reported at realistic concentrations of neonicotinoids in soils and leaf litter, this provides reasonable evidence that some soil ecosystem services can be impaired by the use of neonicotinoid insecticides. Further empirical studies coupled with ecological modeling to test the likelihood and extent of these effects are warranted.

## Freshwater ecosystem functions

### Nutrient cycling and water quality

Pollution by pesticides is widely recognized to be a major threat to freshwater ecosystems worldwide (Gleick et  al. 2001; MEA 2005). Freshwater ecosystems provide an important array of ecosystem services, ranging from clean drinking water and irrigation water to industrial water, water storage, water recreation, and an environment for organisms that support fish and other important foods. Invertebrates make up a large proportion of the biodiversity in freshwater food chains and are a critical link for transfer of energy and nutrients from primary producers to higher trophic levels both in the aquatic and terrestrial ecosystems. Thus, alteration of invertebrate abundance, physiology, and life history by insecticides can have a serious impact on services provided by freshwater ecosystems. Equally, their role in decomposition of organic matter and nutrient cycling offers an essential purification service of water used for human consumption or to support aquatic life.

Peters et  al. (2013) conducted a review of the effect of toxicants on freshwater ecosystem functions, namely leaf litter breakdown, primary production, and community respiration. For the review, 46 studies met their empirical specifications (for example, effect size and control treatment available). An important outcome of their review is that in over a third of the observations, reduction in ecosystem functions was occurring at concentrations below the lower limits set by regulatory bodies to protect these ecosystems. These lower limits were often set using LC_50_ values for common test species like *Daphnia magna*, with risk assessment procedures not including more sensitive species or consideration of species that have critical roles in maintaining ecosystem function. A key shortcoming of the review of Peters et  al. (2013) is that a large number of the included studies involved effects of organophosphates, pyrethroids, and carbamates, but no information is given for the newer insecticide classes such as neonicotinoids or fipronil.

Relatively few studies have formally tested the effects of neonicotinoids or fipronil on ecosystem services in freshwater systems. A recent study by Agatz et  al. (2014) did consider the effect of the neonicotinoid, imidacloprid, on the feeding activity of *Gammarus pulex*, a common freshwater amphipod that plays an important role in leaf litter breakdown. Prolonged inhibition of feeding after exposure was found at concentrations of imidacloprid (0.8 to 30 μg/L) that are within the range of those measured in several aquatic environments. Reduced leaf feeding and altered predator-prey interactions of a similar shredder species, *Gammarus fossarum*, have been reported at thiacloprid concentrations of 1–4 μg/L (Englert et  al. 2012). Similar findings have been shown for other shredder species, stonefly (*Pternonarcyidae*) and crane fly (Tipulidae) larvae, exposed to imidacloprid in leaves and in water exhibiting mortality at 130 μg/L and feeding inhibition at 12 μg/L when applied directly to water but were more tolerant when exposed through the leaves (Kreutzweiser et  al. 2008a). In a second study, the authors were able to determine that the effects on feeding inhibition were important in reducing leaf litter decomposition rates at concentrations of 18 to 30 μg/L (Kreutzweiser et  al. 2009).

Prolonged exposure, or exposure to multiple compounds, might affect this and other shredder populations. Although not widely measured, inhibition of this functional feeding group has the potential to negatively affect the conversion of coarse terrestrial material into fine particulates that can be more readily consumed by other species. This in turn is expected to alter the aquatic invertebrate community, decomposition rates, and nutrient cycling, ultimately influencing water quality and the support of biodiversity which is an important ecosystem service. It should be noted that *G. pulex* is more sensitive to imidacloprid than *Daphnia* species and that both are crustacea and not insects. Several insects tend to be much more sensitive than *G. pulex* to imidacloprid so the risk to decomposition processes might be larger than has been assessed by studies with *G. pulex*, depending on the affected species role in the function of ecosystems and the amount of functional redundancy in the community (Beketov and Liess 2008; Ashauer et  al. 2011).

### Aquatic food chain effects

Ecosystem services related to decomposition and nutrient cycling are important for water quality; however, there is an additional concern for potential indirect effects of insecticides in reducing important invertebrate prey. This may be critical for many freshwater species that are valued for food (e.g., fish and crayfish) and for ecological reasons (amphibians and aquatic birds). While rarely studied, indirect food chain effects have been reported in freshwater systems. For example, Hayasaka et al. (2012a) performed an experimental rice paddy mesocosm study using the systemic insecticides imidacloprid and fipronil, applied at recommended rates. Zooplankton, benthic, and neuston communities in the imidacloprid-treated field had significantly lower species abundance than those from control. Hayasaka et  al. (2012a, b) further found that two annual applications of imidacloprid and fipronil were important in reducing benthic arthropod prey which led to reductions in growth of medaka fish (*Oryzias latipes*). Sánchez-Bayo and Goka (2005, 2006) also studied the ecological changes in experimental paddies treated with imidacloprid throughout a cultivation period. A total of 88 species were observed, with 54 of them aquatic. They reported plankton, neuston, benthic, and terrestrial communities from imidacloprid-treated fields had significantly lower abundance of organisms compared with control. Our knowledge about how aquatic communities react to, and recover from, pesticides, particularly in relation to the water residues, is deficient (Sánchez-Bayo and Goka 2005, 2006).

While not conclusively proven, many of the insectivorous bird species declines are also coincident with agricultural areas using these pesticides and speculation about recent population declines through reductions in emergent invertebrate prey from insecticide use seems plausible given the correlative evidence (Benton et al. 2002; Boatman et  al. 2004; Mason et  al. 2012). Neonicotinoids are the latest generation of pesticides that have the ability to enter freshwater bodies and negatively affect invertebrate populations which in turn can reduce emergent insects that numerous water-dependent birds and other wildlife depend on. A recent study by Hallmann et al. (2014) is the first to demonstrate the  potential cascading effect of low neonicotinoid concentrations in water to insectivorous birds. Future studies should consider the importance of pesticide effects at the community level considering the intricate interaction among species in the trophic chain and the indirect effects on species deemed important for human consumption, recreation, or esthetic value.

### Conclusions on freshwater ecosystem functions

Many aquatic species are directly exposed to neonicotinoid and fipronil insecticides in water, often over prolonged periods. Data from long-term and large-scale field monitoring by Van Dijk et  al. (2013) have demonstrated the negative effects of imidacloprid on invertebrate life. Such negative impacts have the potential to adversely alter the base of the aquatic food web given that this group is a critical link for the transfer of nutrients and energy from primary producers to consumers. Reductions in survival, growth, and reproduction of freshwater organisms, particularly aquatic insects and crustaceans, can alter ecosystem functions related to decomposition and nutrient cycling. These processes are central to providing ecosystem services such as clean freshwater and the support of biodiversity. Equally important are the effects on the trophic structure, which can influence the stability, resilience, and food web dynamics in aquatic ecosystems, but also terrestrial ecosystems given that many aquatic insects have adult life stages out of the water.

## Fisheries and aquaculture

Sustainably managed fisheries and aquaculture can offer solutions to a growing demand for aquatic animal protein sources. In Africa, Asia, and Latin America, freshwater inland fisheries are providing food to tens of millions of people (Dugan et  al. 2010) while ensuring employment, especially to women (BNP 2008). Pesticide use could hamper the successful expansion of global fisheries as well as small-scale inland fisheries, aquaculture, and combined rice-fish farming systems, if those pesticides are negatively affecting fisheries.

Neonicotinoid use has been increasing in fish farming and aquaculture environments because of their relatively low acute toxicity to fish and their effectiveness against sucking parasites and pests. For example, imidacloprid (neonicotinoid) is replacing older pesticides, such as pyrethroids to control rice water weevil (*Lissorhoptrus oryzophilus* Kuscel) infestations in rice-crayfish (*Procambarus clarkii*) rotations (Barbee and Stout 2009) and carbamates (carbaryl) for controlling indigenous burrowing shrimp on commercial oyster beds in Washington (USA) (Felsot and Ruppert 2002). In both of these cases, nontarget effects of imidacloprid to the main fishery have been demonstrated. The degradation of water quality by neonicotinoid pesticides and the resulting ecotoxicological impacts on aquatic organisms are among those risks considered here.

### Threats to cultured fish stocks

The majority of insecticides can affect cultured fish production and other nontarget animals in rice paddy systems. Several wild fish species inhabit the paddy and adjacent drains (Heckman 1979) and can be subjected to the effects of pesticides applied routinely. Fish may be affected indirectly by reductions in food resources, particularly aquatic invertebrates (Sánchez-Bayo and Goka 2005, 2006; Hayasaka et  al. 2012a, b). Although known to have higher lethal tolerance to neonicotinoids, fish can be exposed to sublethal concentrations and their accompanying surfactants, which can cause adverse effects. Imidacloprid was shown to cause a stress syndrome in juvenile Japanese rice fish (medaka). As often happens with stressed fish, a massive infestation by a parasite, *Trichodina ectoparasite*, was observed in medaka fish in imidacloprid-treated fields (Sánchez-Bayo and Goka 2005). In a recent study, Desai and Parikh (2013) exposed freshwater teleosts, *Oreochromis mossambicus* and *Labeo rohita*, to sublethal concentration (LC_50/10_ and LC_50/20_) of imidacloprid for 21 days and found significant alterations in several biochemical parameters (ALT, AST, ALP, and GDH). Increased enzyme activity in tissues indicated liver damage, which the authors concluded, was linked to imidacloprid exposure.

While acute mortality of fish from the neonicotinoid insecticides is rare, Rajput et  al. (2012) reported that imidacloprid was toxic to freshwater catfish, *Clarias batrachus*, when exposed for 21 days, but only at high doses. Protein loss was reported when exposed to high concentrations that later caused lethality. Although this catfish has the potential to become a particularly harmful invasive species in some areas, it is also considered to be one of the most important catfish species in aquaculture given its economic value as food for human populations throughout most of India.

### Shellfish aquaculture

Studies of shellfish aquaculture where neonicotinoids and fipronil are in use are rare. Dondero et  al. (2010) reported negative sublethal effects of imidacloprid and thiacloprid at the transcriptomic and proteomic levels in the marine mussel, *Mytilus galloprovinciali*. In the Willapa Bay (Washington State, USA), imidacloprid is applied directly to exposed sediments, when the tide is out, to control native species of burrowing shrimp (*Callianassa* sp.; *Upogebia* sp.) that can negatively affect oyster production, but its effects on nontarget organisms are unknown. According to Felsot and Ruppert (2002), there was a rapid dissipation of imidacloprid from water and it was hypothesized that this could be due to extensive dilution by the tide. However, it was noted that there is a lack of studies concerning its behavior in the wider estuary ecosystem. Environmental monitoring programs are needed to evaluate exposure to salmonids following the treatment of oyster beds. Potential for adverse effects from exposure to nontarget species residing in the bay, such as juvenile Chinook (*Oncorhynchus tshawytscha*) and cutthroat trout (*Oncorhynchus clarki*), is unknown. Neonicotinoids are frequently detected in estuaries among the pollutants found in estuarine areas where oyster farms are located. Although few reports are available, anecdotal data suggest that neonicotinoids are present in estuary environments and might exert effects on cultured shellfish species or the wider ecosystem, but overall, studies to determine impacts are lacking.

### Neonicotinoids in fish-rice ecosystems

The development of rice-fish farming systems has been viewed as a sustainable option for rural development, food security, and poverty alleviation. Rice-fish farming systems still frequently rely on insecticides to protect rice crops against sucking insect pests, although Integrated Pest Management (IPM) practices are recommended to reduce the use of insecticides and their potential negative effects on fish populations. Imidacloprid is known to persist in treated rice paddy waters, demonstrating that it does not completely degrade in this aquatic environment, and in fact, Tišler et  al. (2009) report that imidacloprid concentrations are increasing in rice paddies. Pesticides can move from treated rice field water to natural water bodies (Heong et  al. 1995; Scientific & Technical Review Panel 2012). A study by Elfmann et  al. (2011) in the Philippines showed that pesticides are frequently found in downstream rivers (Scientific & Technical Review Panel 2012). Given their persistent nature, it is likely that neonicotinoid insecticides used in rice paddies will also move to natural waters and downstream reaches.

### Conclusion on risks to cultured fisheries

The nutritional benefits of fish consumption have a positive link to increased food security and decreased poverty rates in developing countries. Reducing access to fish for consumption could have particular impact on human populations living in less developed countries, where there is limited access to sufficient food. In some countries, high protein meat produced by fisheries can become an important low-cost nonstaple food source.

As with many other contaminants that have threatened natural and managed aquatic ecosystems, neonicotinoids and fipronil may offer an additional threat to cultured fish production. To ensure long-term sustainability and food security from fisheries (Pauly et  al. 2002, 2005), the use of persistent and toxic insecticides in or near fish culture systems should be minimized if those insecticides have been shown to pose risk of harm to fish and their prey species. Although fish appear to have a relatively high toxicity threshold to neonicotinoids, indirect and sublethal effects have been observed from exposure to environmentally relevant concentrations of fipronil, imidacloprid, and thiacloprid. While intensive fish farming can provide important food sources, there is potential for combined or synergistic toxicological effects of diverse contaminants, including neonicotinoids, to threaten fish farm species and other aquaculture commodities.

## Biological pest control

### Predators as natural pest control

Invertebrate predator-prey relationships are an important part of many natural and agricultural ecosystems. Diversity and interdependence of species strongly influence shape and complexity of food webs. Food web complexity and especially the presence of predators are important for humans when considering the natural regulation of invertebrate “pests.” Predation (including parasitism) of invertebrate pests by a diverse array of invertebrate and vertebrate predators can be considered an important ecosystem service, often called “biological control” in agricultural systems (Schlapfer et  al. 1999; Wilby and Thomas 2002; Bradley et  al. 2003).

Although only pest species are targeted by the insecticide, both the pest and natural predators can be affected. Often, the pest, however, exhibits life history strategies that allow their populations to recover faster than their predators. Many of the pest predators are insects and, thus, are also sensitive to neonicotinoid insecticides. In Pisa et  al. (2014, this issue), several examples of affected predatory insect species are given but that review is by no means complete. A growing number of studies indicate that predator species and their ecosystem service are at risk when neonicotinoids are used (see reviews by Desneux et  al. 2007 and Hopwood et  al. 2013). Hopwood et  al. (2013) conclude on the basis of more than 40 toxicity studies across a range of biological pest control species that the widespread use of neonicotinoids negatively impacts predatory and parasitoid species that provide much needed biological control of crop pests. Losey and Vaughan (2006) estimated that the value of natural control agents to control native North American pests is about 13.6 billion dollars, which includes pest predators, but also weather and pathogens.

## Pollination

### Pollination as an ecosystem service

Pollination is considered one of the most essential regulating as well as supporting ecosystem services (Kremen et  al. 2007; De Groot et  al. 2010; Vanbergen and the Insect Pollinator Initiative, 2013) and may be considered as a cultural ecosystem service as well (esthetics). Biologically mediated pollination is the active or passive transfer of pollen within or between flowers via invertebrate, mammalian, or avian vectors. It is a critical service for fruit, vegetables, nuts, cotton, and seed crop production among many others for agricultural crops and supports reproduction of wild plant communities (Allen-Wardell et  al. 1998; Aguilar et  al. 2006; UNEP 2010; Ollerton et  al. 2011; Lautenbach et  al. 2012; Vanbergen and the Insect Pollinator Initiative, 2013).

Without pollination, the fecundity of plants is affected, potentially leading to yield losses in cultivated crops and genetic diversity loss or local extinction in wild plants. Crops can be animal-pollinated, wind-pollinated, self-pollinated, or a combination. In many crops that constitute the human diet, pollination is essential for the setting of fruits and seeds; in others, it promotes these processes in varying gradations. Consequently, the measure of yield increase due to pollination in crops varies greatly; some crops not showing a yield increase, while others do not produce fruits or seeds unless pollinated (Richards 2001; Klein et  al. 2007).

There is a growing concern worldwide about the fate of insect-pollinating species and pollinating services (Potts et  al. 2010; Van der Sluijs et  al. 2013; Vanbergen and the Insect Pollinator Initiative, 2013; Pisa et  al. 2014, this issue). A range of environmental changes that are currently taking place worldwide affect populations of wild and managed pollinating species. These include exposure to toxic chemicals, habitat loss and fragmentation, climate change, pathogens, land-use intensification, parasites, and the spread of invasive species and diseases (Steffan-Dewenter et  al. 2002; Tylianakis et  al. 2005; Biesmeijer et  al. 2006; Kuldna et  al. 2009; Potts et  al. 2010; Vanbergen and the Insect Pollinator Initiative, 2013).

Sánchez-Bayo and Goka (2014) demonstrated that field realistic residues of neonicotinoid insecticides in pollen pose high risk to honeybees and bumblebees, while in the field synergisms with ergosterol inhibiting fungicides will further amplify these risks. They found that imidacloprid poses the highest risk to bumblebees (31.8–49 %, probability to reach the median lethal cumulative dose after 2 days of feeding on field realistic dose in pollen) and thiamethoxam the highest risk to honeybees (3.7–29.6 %). Other pollinators were not included in their risk assessment. An increase in AChE activity in honeybees was related to in-field exposure to corn pollen in neonicotinoid seed-treated fields (Boily et  al. 2013). Because of the persistence of neonicotinoids in soil and water and their use as systemics, which facilitate uptake by wild plants and agricultural crops, all pollinators can be exposed to these insecticides at lethal or sublethal concentrations through multiple exposure routes (Van der Sluijs et  al. 2013). Neonicotinoids and fipronil have known lethal and sublethal effects on domestic and wild insect pollinator populations at extremely low concentrations, often reported in the parts per trillion range (Pisa et  al. 2014, this issue).

### Pollination of crops

Pollinating services are provided by managed honeybees (*Apis mellifera*), but also by wild species such as solitary, stingless bees and bumblebees. In addition, flies, butterflies, wasps, moths, beetles, and other invertebrates and, in some cases vertebrates (such as bats, squirrels, birds and some primates), are also known to pollinate natural plants and crops (Buchmann 1997; Klein et  al. 2007; De Luca and Vallejo-Marín 2013; Ghanem and Voigt 2012; Vanbergen and the Insect Pollinator Initiative, 2013). Over 25,000 species of bees have been identified (FAO 2013a), which are responsible for a large portion of pollination services worldwide (Danforth et  al. 2006; Breeze et  al. 2011). In Europe alone, more than 2,500 species of bees are known pollinators (Vaissiere et  al. 2005).

Contrary to popular belief, estimates for the UK indicate that managed honeybees (*A. mellifera*) pollinate approximately one third of the crops, at most (Breeze et  al. 2011). Although debated, there is evidence that numerous wild bee species also contribute substantially to the quality and reliability of pollination of a broad range of crops (e.g., Chagnon et  al. 1993; Bosch et  al. 2006; Greenleaf and Kremen 2006; Hoehn et  al. 2008; Lye et  al. 2011). Wild insect pollinator species are regarded as the most effective pollinators on fruit crops and seem to be more sensitive to pesticides than honeybees (Cresswell et  al. 2012; Laycock et  al. 2012). Economic gain from insect pollination on crops increases significantly with increasing numbers of wild bee species in the European Union (Leonhardt et  al. 2013). In addition, bumblebees (*Bombus* spp.) are the predominant or exclusive pollinators of many wild plant species (Goulson 2003).

### Pollination of wild plants

In addition to pollinating crops, which make up <0.1 % of all flowering plants worldwide, between 60 and 85 % of wild angiosperms (flowering plants) require animal pollinators (Kearns and Inouye 1997; Ashman et  al. 2004). Ollerton et  al. (2011) estimated that 299,200 species (85 %) of angiosperms depend on pollinators worldwide. However, this estimate does not account for the mean proportion of angiosperms per latitude, varying from 78 % of species in temperate zones up to 94 % in tropical regions. Vanbergen and the Insect Pollinator Initiative, (2013) estimated that insects enable reproduction globally for up to 94 % of wild flowering plants. Pollination of wild plants contributes to human welfare indirectly, of which some examples are esthetics of the landscape, the pleasure of looking at foraging bumblebees in richly flowering meadows, and providing forage for wildlife (Jacobs et  al. 2009). Pollination is also instrumental in increasing the genetic diversity in plant species (Benadi et  al. 2013).

The impact of insect pollinator loss on ecosystem function is not well understood, although a few cases have been described. An example of a subtle but important interaction is the one between wild species and honeybees. Greenleaf and Kremen (2006) studied pollinator efficiency of honeybees on sunflowers and discovered a fivefold increase in efficiency in the presence of wild bees. Such phenomena are likely to occur in natural environments as well, meaning that the loss of one species can radically alter pollination dynamics of wild plants in affected communities. Furthermore, knowing that the survival of certain host plants is directly linked to the survival of their pollinating species (Kim 1993), this can have a knock-on effect in the biotic community. For instance, Kearns and Inouye (1997) describe how keystone species such as fig trees, one of the 750 species often dependent on a distinct and unique wasp species for pollination, provide the staple food for many species of vertebrate wildlife in tropical communities. The loss of these wasps has the potential to lead to a complete shift in biotic community structure of these areas. The same goes for other areas with specialized pollinator-plant interactions, such as South Africa (Ollerton et  al. 2011).

Although wild plants are often dependent on multiple pollinators or may be able to use wind pollination, it is important to realize that pollinating insects fulfill a crucial role in the ecological food webs. Loss of pollinating species can also affect other networks, thus leading to impairment in ecosystem functioning as a whole (Bartomeus et  al. 2013; Burkle et  al. 2013; Labar et  al. 2013).

### Conclusions on ecosystem services from pollinators and other beneficial insects

The role of insects as consumers, predators, pollinators, and decomposers in ecosystems is critical for ecosystem function. High sensitivity of many key pollinating and predating insect species to neonicotinoids, combined with the high risk of exposure, raises concerns about the (long-term) impact of these substances. Adverse impacts of wide-scale insect pollinator and predator loss include cascade effects in biotic communities that can ultimately affect human populations. In human dimensions, the ecosystem services pollination and biological control together represent an estimated global value of about US$215 billion in 2005 (Vanbergen and the Insect Pollinator Initiative, 2013). The global loss of bee species, as bioindicators of environmental health, is an early warning that global biodiversity and ultimately, human welfare, may be threatened.

## Food security

### Pollinator-dependent crops

Although the estimated percentage of human food that depends on bee-pollinated crops is relatively small, 15–30 % (O’Toole 1993, in Kearns and Inouye 1997; Greenleaf and Kremen 2006), important components of food production, diversity, security, and stability rely on animal pollinators (Steffan-Dewenter et  al. 2002, 2005). Of the 124 major commodity crops directly used for human consumption, 87 (70 %) are dependent on pollination for enhanced seed, fruit, or vegetable production. These 87 crops are essential to our quality of life providing the quality and diversity of the vegetables and fruits we eat and amount to 23 × 10^8^ megatons (35 %) of global food production volume, although only part of this amount is directly attributable to pollination (Klein et  al. 2007).

Roubik (1995, in Klein et  al. 2007) provided a list of 1,330 tropical crops, of which ca. 70 % have one or more varieties that show improved production after animal pollination. More specifically, for the European situation, 84 % of crop species produced depend on pollination (Williams 1994), with a total of 12 % of the total cropland area dependent on pollination (Schulp et  al. 2014).

The relative importance of crop pollination as an ecosystem service is increasing worldwide. In 2006, pollinator-dependent crops contributed 16.7 and 9.4 % more to total agricultural production in the developed and developing world, respectively, than in 1961 (Aizen et  al. 2008; Aizen and Harder 2009). Since then, the continued and foreseen increase in the production of pollinator-dependent crops such as oil palm, sunflower, and canola (FAO 2013b; Schulp et  al. 2014) indicates a further rise in these percentages.

### The economic value of pollination

The economic value of pollination services can be considered to be the marginal increase in plant production due to pollination (Kremen et  al. 2007), for those plants that have a market or subsistence value to humans. Examples are crops used for food or feed, timber, or fiber. Therefore, the loss of insect pollinators has large potential consequences on human food production directly through reduced crop yields. Richards (2001) provides a good overview of impacts on crop yield through inadequate pollinator service. Although pollinator decline was not documented to affect crop yield on a global scale in 2008 (Aizen et  al. 2008), there is evidence on a local scale that declines in pollinator (diversity) affect fruit set and seed production (Brittain et  al. 2013). The absence of pollinators thus would translate into a 7 % drop in crop production in the EU (Schulp et  al. 2014). These crops are nonetheless those that bring our diversity of food in civilized societies and quality of life (Klein et  al. 2007).

A second impact of pollinator loss is the reduced production of crops that become less valued by the consumer and are therefore sometimes nonsaleable. Some examples are cucumbers and apples, of which the fruits do not grow according to market standards without proper pollination. Lack of pollination will reduce their value or render them worthless (e.g., curled cucumbers, lopsided apples) (Morse and Calderone 2000).

Increased production costs are a third potential impact of pollinator loss. Almond farmers in the USA, which are completely dependent on commercial pollination services, have experienced a sharp increase in the price for crop pollination services since 2005, due to pollinator scarcity (Sumner and Boriss 2006; Carman 2011).

Many animal-pollinated crops are locally important for the economy of the region. Some examples are olives, sunflowers, and cotton that are not wholly dependent on pollinators, but production is enhanced. Several crops that are completely dependent on pollination are often specialty products that are not sold on a large scale, such as vanilla (Richards 2001), but are nonetheless an essential resource to specific regions.

Several national studies (e.g., USA: Morse and Calderone 2000; Losey and Vaughan 2006) have applied dependence ratios per crop type, calculating the actual impact on crop production in the absence of pollinators. Although a potentially useful tool, the ratios that were used varied widely between studies and regions. Gallai et  al. (2009) therefore aimed to provide an economic valuation of complete world insect pollinator loss, including economic vulnerability per region. The authors calculated a value of €153 billion, 9.5 % of the total value of crops produced globally for direct human consumption in 2005. In the EU, pollinator-dependent crops currently represent 31 % of the EU income from crop production. The total monetary value for insect-pollinating services therein is between 10 and 12 % (Leonhardt et  al. 2013; Schulp et  al. 2014).

### Food supply and food quality

With the expected population growth in the coming decades, meeting the increasing food supply needs in a sustainable way will become a major challenge. The environmental consequence of the intensification of agricultural systems may pose a threat to the future accessibility to an adequate food supply (Matson et  al. 1997). But beyond securing access to sufficient food for all people, the need to provide a supply of safe and nutritionally high-quality food to achieve a balanced diet has become an important consideration in order to avoid health impacts such as intellectual and physical disabilities. Access to a large diversity of fruit and vegetables also contributes to the enjoyment of quality foodstuff and food culture that contributes to overall social and cultural identity.

The capability of responding to the current human nutrient requirements is crucial, according to the World Health Organization (WHO 2006). Many people are affected by vitamin and mineral deficiencies, especially in developing countries where one out of three persons suffer from chronic undernourishment in energy and in micronutrients (vitamins and minerals). Eilers et  al. (2011) studied the proportion of nutrients derived from more than 150 global leading crops and found that although minerals seem to be fairly evenly distributed over crop types, certain vitamins are scarcer in pollinator-independent crops. An example is the carotenoid group, in which 99.33 and 100 % of β-cryptoxanthin and lycopene, respectively, are provided by pollinator-dependent crops.

In contrast, the developments in agriculture worldwide have largely increased the production of staple foods such as potato, cassava, corn, rice, and wheat over the last 25 years (FAO 2013b). These staple crops are mostly wind- or self-pollinated or propagate otherwise, so do not depend on pollination services. Although these crops provide the required caloric intake, they contain relatively low levels of most micronutrients. Globally, more than two billion people are affected by “hidden hunger,” a micronutrient deficiency caused by poor diet diversity (Welch and Graham 1999; Muthayya et  al. 2013). Pollinator losses leading to reduced diet diversity, especially from plants that provide a larger array of micronutrients, may exacerbate the negative impact on health and economic development in certain regions.

### Seed security and seed treatments

Seed security is seen as a key driver of food security (Sperling and McGuire 2012). Food production agronomic traits such as yield, early maturity, resistance to specific stresses, and also nutritional traits should be among the diverse goals of seed security (Sperling and McGuire 2012). Agroecosystems of even the poorest societies have the potential through ecological agriculture and IPM to meet or even exceed conventional yields produced by conventional methods and supply regional and international markets across the developing country regions (IAASTD 2009).

The increased and often prophylactic use of neonicotinoid seed-coated hybrids cannot be viewed as a sustainable way to protect crops from insect damage given the risks described to pollinators, soil organisms, and aquatic invertebrates. Seed treatments offer an easy incentive to farmers to act as a form of crop protection insurance by applying a treatment in anticipation of the pest problem. However, in order for this technique to be ecologically, economically, and socially viable, substantial gains must be seen in yields to offset risks to ecosystem health. In Britain, as elsewhere, agricultural practices have seen rapid increases in the use of neonicotinoid-treated seeds over the past decade. However, little or no gains have been observed in crop yields over the same period or those gains were not great enough to offset the cost of the seed treatment (Goulson 2013). For example, in Canada’s Prairie region, canola (oilseed rape) crops cover 8.5 million hectares of cropland, and 95 % of the canola seeded is coated with neonicotinoids (Main et al. 2014). The authors conservatively estimated that neonicotinoid use in that region of Canada amounted to 44 % of the cropland in a single year or 215,000 kg. Systemic seed treatments have facilitated the extended and widespread use of neonicotinoid insecticides in modern agriculture and represent a threat to agrobiodiversity and food security.

### Insecticide resistance

Several crop pests have begun to develop pesticide resistance to neonicotinoids (Jeschke et  al. 2011). Examples are imidacloprid and acetamiprid resistance in cotton aphids (*Aphis gossypii*) (Herron and Wilson 2011). Other crop pests that show neonicotinoid resistance are the Greenhouse whitefly (*Trialeurodes vaporariorum*) (Karatolos et  al. 2010) and the Colorado potato beetle (*Leptinotarsa decemlineata*) (Szendrei et  al. 2012).

The development of insecticide resistance has also been reported for the brown planthopper (*Nilaparvata lugens*) in East Asian countries such as Vietnam, China, and Japan (Wang et  al. 2008). Planthopper resistance to imidacloprid was reconfirmed in more recent studies (Azzam et  al. 2011). Zhang et  al. (2014) studied nine field populations of the brown planthopper (*N. lugens*) from Central China, East China, and South China, and resistance to insecticides was monitored from 2009 to 2012. All nine field populations collected in 2012 had developed extremely high resistance to imidacloprid, with resistance ratios ranging from 209.3 to 616.6. Resistance to neonicotinoids was much higher in 2012 than in 2009. The resistance ratio of thiamethoxam varied from 17.4 to 47.1, and the resistance ratio of nitenpyram varied from 1.4 to 3.7 in 2012. Of the nine field populations, six populations showed higher resistance to nitenpyram in 2012 than in 2011. Taken together, these reports demonstrate that the widespread use of neonicotinoids increases the rate of the development of target pest resistance. Insect resistance, in turn, usually results in increased application rates or frequency of an insecticide, leading to greater economic and environmental costs.

### Conclusions on food security

The definition of food security within the United Nations framework includes the physical availability of food and its stability over time (FAO 2008). Quality and diversity of food and the ecological and social sustainability of the food production are also important parts of food security. Agriculture is becoming more pollinator dependent because of an increasing consumption of pollinator-dependent crops (Aizen et  al. 2008). Neonicotinoid insecticides are recognized to be a threat to domestic pollinators such as honeybees but also many wild pollinator species. Although theoretically possible, a global decrease in crop yields and diversity of fruit and vegetables due to reductions in pollination has not yet been demonstrated, but evidence exists at regional scales. Widespread use of seed treatments does not necessarily increase crop yields, but appears to be threatening pollinator and soil health as well as promoting insect pest resistance. Extensive and wide-scale use of any single insecticide has the proven potential to become a threat to agrobiodiversity.

Agrobiodiversity can be thought of as the outcome of agricultural practices that produce a variety of crops, including those that provide essential micronutrients. The focus of future agriculture should not be limited to an increase in overall production, but should also consider the maintenance of genetic diversity in crop plants, which provide valued agronomic traits (Sperling and McGuire 2012). The preservation of agrobiodiversity and seed security will be achieved by promoting varieties of crops already known in the area, making local (traditional) nutritious varieties more accessible. Many of these crops depend on insect pollination and are therefore at risk from widespread and persistent use of insecticides that negatively affect pollinators. In this regard, the use of neonicotinoid insecticides may threaten food security and the development of sustainable agriculture.

## Conclusions

In this paper, we examine the potential impact of systemic insecticides, particularly neonicotinoids but also fipronil, on a variety of ecosystem functions and services. The paper explores the role and vulnerability of invertebrates in soil function and food production systems, as well as threats to the aquatic biodiversity that supports cultured fisheries. Clear evidence of the critical role of microbes, insects, and other invertebrates as consumers, predators, pollinators, and decomposers for the maintenance of healthy ecosystem functions and food production is presented. In exploring the indispensability of these organisms, their vulnerability to systemic insecticides has been highlighted. Most neonicotinoid insecticides are persistent in soil and water and can be found in dust particles during sowing of dressed seeds and are therefore likely to encounter and potentially affect a broad range of biological organisms that provide ecosystem services.

Neonicotinoid and fipronil pesticides are bioavailable in the environment at levels that are known to cause lethal and sublethal effects on a wide range of terrestrial, aquatic, and soil beneficial microorganisms, invertebrates, and vertebrates. These beneficial organisms possess a diversity of traits (e.g., nitrogen fixers, pollinators, and nutrient recyclers) that are key to healthy ecosystem functioning and services (Perrings et  al. 2010). There is increasing evidence that the widespread use of neonicotinoids and fipronil is causing harm to these beneficial organisms, and therefore, those impacts have the potential for reducing ecosystem services, either consumptive (e.g., food, fuel) or nonconsumptive (e.g., health).

To help feed the world’s population adequately, crop protection methods and products will always be needed to reduce yield losses caused by pests. But sustainable choices should be made while implementing pest control methods and products in order to alleviate potential harm for food security, ecosystem services, and the full functionality of all systems of the environment. Relying on pesticide tolerance and the selection of resistance traits and/or a functional resilience of ecosystems’ communities (Köhler and Triebskorn 2013) as justification for the continued widespread and often prophylactic use of neonicotinoid and fipronil insecticides would be a perilous strategy for maintenance of ecosystem services. While the link between nontarget impacts of these systemic insecticides and their effects on ecosystem services is not always clear in the published literature, their widespread use, persistent nature, and toxicity to a broad range of beneficial organisms are strong indications that ecosystem services dependent on these organisms may be at risk.
